# The Effect of Polymer–Solvent Interaction on the Swelling of Polymer Matrix Tablets: A Magnetic Resonance Microscopy Study Complemented by Bond Fluctuation Model Simulations

**DOI:** 10.3390/polym16050601

**Published:** 2024-02-22

**Authors:** Franci Bajd, Urša Mikac, Aleš Mohorič, Igor Serša

**Affiliations:** 1 Jožef Stefan Institute, 1000 Ljubljana, Slovenia; franci.bajd@ijs.si (F.B.); ursa.mikac@ijs.si (U.M.); ales.mohoric@fmf.uni-lj.si (A.M.); 2 Department of Physics, Faculty of Mathematics and Physics, University of Ljubljana, 1000 Ljubljana, Slovenia; 3 Institute of Anatomy, Faculty of Medicine, University of Ljubljana, 1000 Ljubljana, Slovenia

**Keywords:** polymer matrix hydration, swelling, magnetic resonance microscopy, Monte Carlo, bond fluctuation model, correlation function

## Abstract

Polymer matrix tablets are an important drug-delivery system widely used for oral drug administration. Understanding the tablet hydration process, both experimentally and theoretically, is, thus, very important for the development of drug delivery systems that exhibit high drug loading capacity and controlled release potential. In this study, we used magnetic resonance microscopy (MRM) to nondestructively and dynamically analyze the water hydration process of xanthan-based tablets. The swelling process was characterized by well-resolved fronts of erosion, swelling, and penetration. The experimental results were complemented by numerical simulations of the polymer matrix hydration process. In the simulations, the polymer tablet matrix was modeled as an assembly of interacting chains with embedded drug particles, while its hydration process was mediated by interaction with solvent particles. The swelling dynamics were modeled within a Monte Carlo-based bond fluctuation model (BFM) that elegantly accounted for steric and nearest-neighbor interactions. This study provides an efficient experimental–theoretical approach for the study of polymer matrix swelling processes.

## 1. Introduction

Polymer matrix tablets are an important drug-delivery system that is widely used for oral drug administration [1]. Basically, these tablets are composed of two major components, i.e., a polymer matrix carrier that swells upon exposure to the solvent environment, and the embedded drug molecules that are gradually released from the pores of the swelling polymer matrix [2]. Microscopically, the tablet matrix consists of an assembly of mutually entangled and interconnected individual polymer chains, of which spatial distribution provides the matrix scaffold. The tablet structure thus exhibits the sites with locally increased polymer density as well as interstitial vacancies that are available for drug molecules [3]. The structure is highly dependent on the concentration and the spatial distribution (heterogeneity) of the involved compounds as well as on their mutual interactions [4]. The pairwise interaction strengths between the tablet compounds and the solvent molecules are also crucial parameters that determine the dynamics of the tablet hydration process and, thus, the drug release kinetics [5]. Specifically, the presence of solvent molecules imposes an imbalance in the interaction pattern, which results in energetically favorable particle rearrangements. The rearrangements are macroscopically manifested in the form of solvent penetration into the tablet interior and concomitant swelling of the polymer matrix that is followed by controlled drug release [6]. Polymer matrix layers with a sufficient amount of up-taken solvent undergo a glassy-to-rubbery phase transition [7], resulting in a polymer matrix expansion, while the still-dry tablet core remains structurally unchanged. Traditionally, the tablet swelling dynamics can be characterized by three characteristic fronts [8], i.e., the erosion front (EF) between the completely swollen polymer matrix and the solvent medium, the swelling front (SF) defining the border between the tablet core in a glassy state and the swollen matrix in a rubbery state, as well as the penetration front (PF) that is determined by the penetration reach of the solvent molecules.

In recent years, different imaging techniques have been successfully employed to dynamically monitor the hydration process of polymer matrix tablets, such as Fourier transform infrared spectroscopic imaging and UV–visible spectroscopy [9,10,11,12], as well as magnetic resonance imaging (MRI) [13,14,15,16]. While the light-based techniques provide high spatial resolution and also enable the spectroscopic characterization of the tablet’s superficial layers that are limited by the light penetration depth, MRI enables noninvasive tomographic characterization of the entire tablet structure, but with a comparatively larger voxel size. Therefore, experimental studies of tablet swelling that employ various complementary techniques to cover different spatiotemporal scales are commonly combined with a number of different mathematical modeling approaches [17]. These approaches differ by their complexity and, hence, also by their predictive power. For example, the empirical models [18] with few fitting parameters are typically applied only in the context of an approximate characterization of the swelling process, while the mechanistic models, implementing transport equations of all involved compounds, provide a more efficient framework for the characterization of tablet swelling with a higher predictive potential. The later models are capable of also accounting for the system heterogeneity. However, they consequently also pose higher computational demands and implementation challenges. For example, Goepferich devised a stochastic coarse-grained model for the simulation of bulk polymer matrix degradation [19], in which discrete polymer matrix volume elements (pixels) were degraded according to the experimentally expected lifetime. The results of this model agreed well with the experimental results, as it correctly accounted for percolation phenomena and the spontaneous mass loss of the dissolving polymer matrix. Therefore, the model influenced the development of other diffusion-controlled and erosion-controlled mathematical models [20,21,22,23]. Other mechanistic models applied to polymer matrix swelling are mesh-free [24] molecular dynamics (MD)-based models [25] and finite element models (FEM) [26] that consider a dissolving polymer matrix and surrounding solvent medium either as atoms or as continuous media, respectively. An efficient computational approach for polymer dynamics simulations is also a bond-fluctuation model (BFM) [27,28], which is a stochastic Monte Carlo (MC) self-avoiding model. In the model, monomers constituting polymer chains have a finite size and MC-attempted moves, providing bond fluctuation in the form of both bond elongations and shortenings, are limited to a certain number of allowed bond lengths. In further studies, Sommer et al. applied the explicit-solvent BFM model to study the effect of inter-particle interactions on the translocation of homopolymers [29] or triblock copolymers [30] through a selectively permeable lipid bilayer. Moreover, the model was successfully applied to study adsorbed/grafted [31], dendritic polymer structures [32] in relation to inter-particle interaction energies and translational dynamics of chain-like particles through mucosal scaffolds [33]. However, the BFM model was only rarely applied in the context of controlled drug release [34].

Compared to [34], where the dynamics of a single polymer chain through a pore were studied, this study presents experimental results of tablet dissolution complemented by numerical simulations using the BFM model with a more complex set of system subcomponents, such as polymer chains, solvents, and drug molecules. Magnetic resonance microscopy (MRM) was used to dynamically follow a water hydration process of xanthan polymer matrix tablets. The experimental results were characterized in terms of the three characteristic tablet fronts (erosion, swelling, and penetration fronts). The experimental results were then complemented by the results of explicit-solvent 2D BFM simulations [35], in which solvent-mediated polymer matrix hydration was studied for different solvent densities and various pairwise interaction strengths between the involved chemical species. Both results, experimental and simulation, confirm the effect of polymer–solvent interaction on polymer matrix swelling dynamics.

## 2. Materials and Methods

### 2.1. Experimental Section: Magnetic Resonance Microscopy (MRM)

For the swelling and drug release studies, cylindrical flat-faced tablets composed of 300 mg of xanthan with molecular weight (MW) of 2 × 10^6^ g/mol (Sigma-Aldrich Chemie, Taufkirchen, Germany) and 100 mg model drug pentoxifylline with MW = 278.31 g/mol (Krka d.d, Novo mesto, Slovenia) were used. The tablet swelling was studied in purified water with ionic strength μ = 0 M. The tablet swelling was monitored by magnetic resonance imaging (MRI) using a Tecmag Apollo (Tecmag, Houston, TX, USA) MRI spectrometer with a superconducting 2.35 T horizontal bore magnet (Oxford Instruments, Oxon, UK) equipped with gradients and RF-coils for MRM (Bruker, Ettlingen, Germany). The tablet was inserted in a container so that only upper horizontal circular flat surface was exposed for the medium penetration. To follow the penetration, swelling, and erosion front three, different MRM methods were used. The position of the penetration front was followed by 1D single point imaging (SPI) sequence with an encoding time *t*_p_ = 0.17 ms, a radiofrequency excitation pulse of 20°, and a repetition time (TR) of 200 ms. The swelling point was determined from 1D SPI *T*_2_ mapping sequence with the same acquisition parameters.

**Figure 1:**
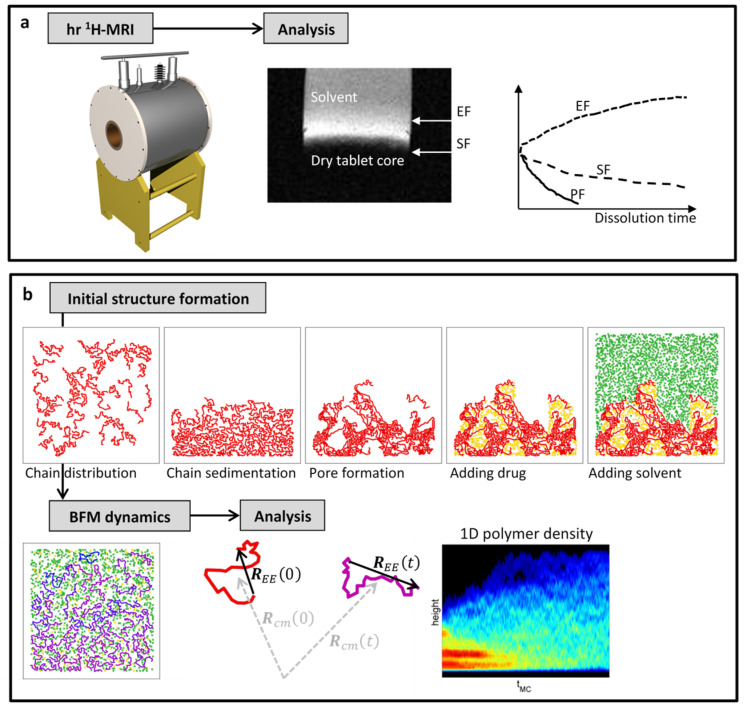
A flowchart schematically summarizing the essential steps in (**a**) magnetic resonance imaging (MRI) experiments of polymer tablet swelling enabling the determination of erosion, swelling, and penetration fronts (EF, SF, PF) and in (**b**) explicit-solvent 2D bond fluctuation model (BFM) simulations of the swelling process of drug-containing polymer matrices.

As for 1D SPI sequence, and by varying the inter-echo time of the preparation CPMG train from 0.3 to 10 ms, the field of view (FOV) of the SPI sequences was 45 mm with a resolution of 350 μm. The erosion front position was measured by 2D *T*_1_-weighted spin-echo sequence, with an echo time (TE) of 6.2 ms and TR of 200 ms. The FOV of the 2D sequence was 50 mm with in-plane resolution of 200 μm and slice thickness of 3 mm. After the medium was added to a tablet, all three MRM measurements were repeated every 30 min for 24 h. A schematic presentation of the experimental setup is depicted in Figure 1a.

### 2.2. Numerical Simulations: Bond Fluctuation Model (BFM)

A polymer matrix was modeled as an assembly of monodisperse 2D BFM chains ($N_{c}$ chains, each with $N_{m}$ monomers), while solvent ($N_{s}$) and drug ($N_{d}$) molecules were modeled as 2D BFM monomers. The dynamics of the involved compounds (chains, solvents, and drugs) were calculated according to the 2D BFM dynamics scheme [35]. In this scheme, an attempted move of a selected monomer with a step size equal to a unit size in a Cartesian lattice was accepted according to Metropolis Monte Carlo (MC) algorithm [36], in which the probability of acceptance, $P=\min\left\{1,\exp\left(-\Delta E\right)\right\}$, was governed by the move-related energy difference ΔE. For the chain monomers, an additional BFM-imposed constraint was considered, in which the attempted monomer moves were accepted, if the final bond lengths between the adjacent chain monomers were equal to either 2, $\sqrt{5}$, $2\sqrt{2}$, 3, $\sqrt{10}$, or $\sqrt{13}$ [35]. In the model, strong steric interactions between the involved species, which were implemented as $E_{steric}\to \infty$, thus resulting in MC-based rejection, provided self-avoidance of fluctuating polymer chains, as well as prevented possible overlaps between monomers. The model also accounted for, in general six, nearest-neighbor (NN) pairwise interactions, $E_{cc}$, $E_{cd}$, $E_{cs}$, $E_{dd}$, $E_{ds}$, and $E_{ss}$ for chain–chain, chain–drug, chain–solvent, drug–drug, drug–solvent, and solvent–solvent interactions, respectively. Pairwise interactions were nonzero only when the interaction pair was in contact.

Initially, the polymer matrix was generated by evenly distributing globular polymer chains exhibiting only the two shortest 2D BFM allowed bond lengths, i.e., 2 and $\sqrt{5}$, across the empty simulation box. The reduced bond length set enabled relatively fast population of the simulation box for high values of $N_{c}$ and $N_{m}$. The polymer chains were then sedimented at the bottom of the simulation box. The sedimentation was achieved in 10^6^ attempted MC moves of two types, individual monomer moves and center-of-mass moves of individual polymer chains. The moves were accepted with the probability $\min\left\{1,\exp\left(-\Delta E_{j}\right)\right\}$, where the potential energy of the selected (*j*th) entity (monomer or chain) was proportional to its vertical coordinate, $E_{j}=\frac{y_{j}}{2h_{y}}$, where $y_{j}$ and $h_{y}$ are its vertical coordinate and simulation box height, respectively. The initial polymer matrix was finalized in the pore formation process, in which polymer dynamics was performed in 10^6^ attempted MC moves with $E_{cc}=-2.0$ (in relative units). This resulted in a heterogeneous polymer matrix structure exhibiting nucleation nodes with an increased polymer density and complementary interstitial vacancies. After calculating the tablet’s superficial layer, the polymer matrix below and the empty space above the layer were populated by $N_{d}$ drug and $N_{s}$ solvent BFM monomers, respectively, in order to obtain a drug-containing polymer matrix tablet immersed in the solvent environment. Drug and solvent monomers were randomly distributed in corresponding spaces by considering the steric interaction and disregarding the nearest-neighbor interaction.

The tablet swelling process was simulated by assuming initially immobile polymer chains. The 2D BFM dynamics of the chain were activated upon the first NN contact with a solvent monomer. This enabled initiation of the swelling process that was initially limited to the tablet’s superficial layers and later progressed to the matrix interior. In the swelling process, only two NN pairwise interaction energies, i.e., chain–chain $E_{cc}=\varepsilon_{0}H$ and chain–solvent $E_{cs}=\varepsilon_{0}\left(1-H\right)$, were varied, while the other four pairwise NN energies were set to zero ($E_{cd}=0$, $E_{dd}=0$, $E_{ds}=0$, and $E_{ss}=0$), in order to keep the 2D BFM simulations sufficiently parsimonious. Here, $\varepsilon_{0}=0.8$ is dimensionless reference interaction energy and H is a parameter that is, in the context of a symmetrized interaction scheme for a chain–solvent-lipid BFM model [29] referred to as relative hydrophobicity. The tablet swelling process was simulated for 10^9^ attempted MC moves. The instantaneous coordinates of the 2D BFM system, intended for subsequent quantitative analyses and snapshot visualization, were stored after every 10^5^ attempted MC moves.

For all three involved species, the swelling process was subsequently analyzed by means of 1D density profiles as a function of time: swelling polymer chains $\rho_{c}^{1D}\left(t\right)$, releasing drug $\rho_{d}^{1D}\left(t\right)$, and penetrating $\rho_{s}^{1D}\left(t\right)$. Moreover, the polymer dynamics were also analyzed in terms of normalized orientational correlation function [37]:

(1) $$C_{EE}\left(t\right)=\frac{\langle R_{EE}\left(t\right)\cdot R_{EE}\left(0\right)\rangle }{\langle R_{EE}\left(0\right)\cdot R_{EE}\left(0\right)\rangle },$$

between the instantaneous, $R_{EE}\left(t\right)$, and initial, $R_{EE}\left(0\right)$, end-to-end chain vectors, as well as in terms of translational correlation function [38]:

(2) $$\Delta R_{cm}^{2}\left(t\right)=\langle {\left(R_{cm}\left(t\right)-R_{cm}\left(0\right)\right)}^{2}\rangle ,$$

between the instantaneous, $R_{cm}\left(t\right)$, and initial, $R_{cm}\left(0\right)$, center-of-mass polymer chain vector. Here, the symbol 〈…〉 denotes an assemble average over the solvent-activated polymer chains. The calculated correlation functions, Equations (1) and (2), were further modeled by empirical relationships:

(3) $$C_{EE}\left(t\right)=e^{-t/\tau }$$

(4) $$\Delta R_{cm}^{2}\left(t\right)=\alpha \;t^{\beta },$$

where τ (orientational correlation time), α, and β (translational correlation exponent) are fitting parameters. In addition, translational correlation function $\Delta R_{cm}^{2}\left(t_{f}\right)$ evaluated at the final simulation time was also calculated. The final simulation time $t_{f}$ was defined as the number of attempted MC moves $T_{dis}$ per the total number of BFM monomers $N_{d}+N_{s}+N_{c}N_{m}$, i.e., $t_{f}=T_{dis}/\left(N_{d}+N_{s}+N_{c}N_{m}\right)$ [29]. A flowchart of the 2D BFM simulations is presented in Figure 1b.

The 2D BFM simulations, along with the analyses, were performed by using in-house written software that was implemented within the Matlab programming environment (Mathworks, Natick, MA, USA). The performance of the simulations was further improved by employing a C/C++-based Matlab executable (MEX) approach. Moreover, calculation of the pairwise interaction was optimized by employing Verlet list approach [39] that was updated after each 100 attempted MC moves. With $N_{c}=45$, $N_{m}=30$, $N_{d}=200$, and $N_{s}=1000$, each simulation run performed on a single processor core (2.9 GHz Intel i7) took approximately 10 h. The instantaneous snapshots of 2D BFM particles were visualized by using the PovRAY rendering program (Persistence of Vision Pty. Ltd., Williamstown, Victoria, Australia).

## 3. Results

**Figure 2:**
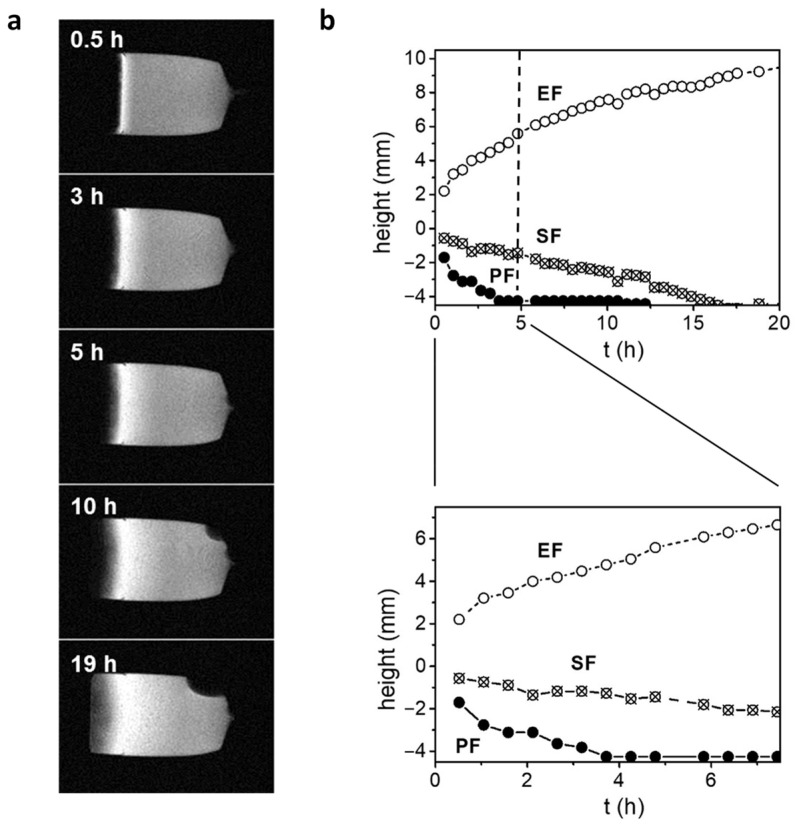
Experimental results: (**a**) representative central-slice MR images of polymer matrix tablets during swelling in tap water and (**b**) the corresponding three characteristic tablet swelling fronts: erosion front (EF), swelling front (SF), and penetration front (PF) as functions of time.

This study combines the methodology of our previous MR microscopy studies on the hydration process of xanthan polymer matrix tablets, e.g., Mikac et al. [14], and uses it to obtain sequential images of the process, which are later used to calibrate and verify the simulation of the same process. An example of these images is shown in Figure 2, along with plots of the positions of the penetration, swelling, and erosion fronts during the swelling of a xanthan tablet. The latter were determined with 1D SPI, 1D SPI *T*_2_ mapping, and 2D spin-echo sequences, as described in [14]. The position of the penetration front moves into the tablet with time and reaches the bottom of the tablet in less than 4 h. The transition from glassy to rubbery state (hydrogel) is delayed and the swelling front reaches the lower edge of the tablet after 15 h. The position of the erosion front moves out of the tablet as the xanthan polymer chains relax and finally separate from the gel layer.

**Figure 3:**
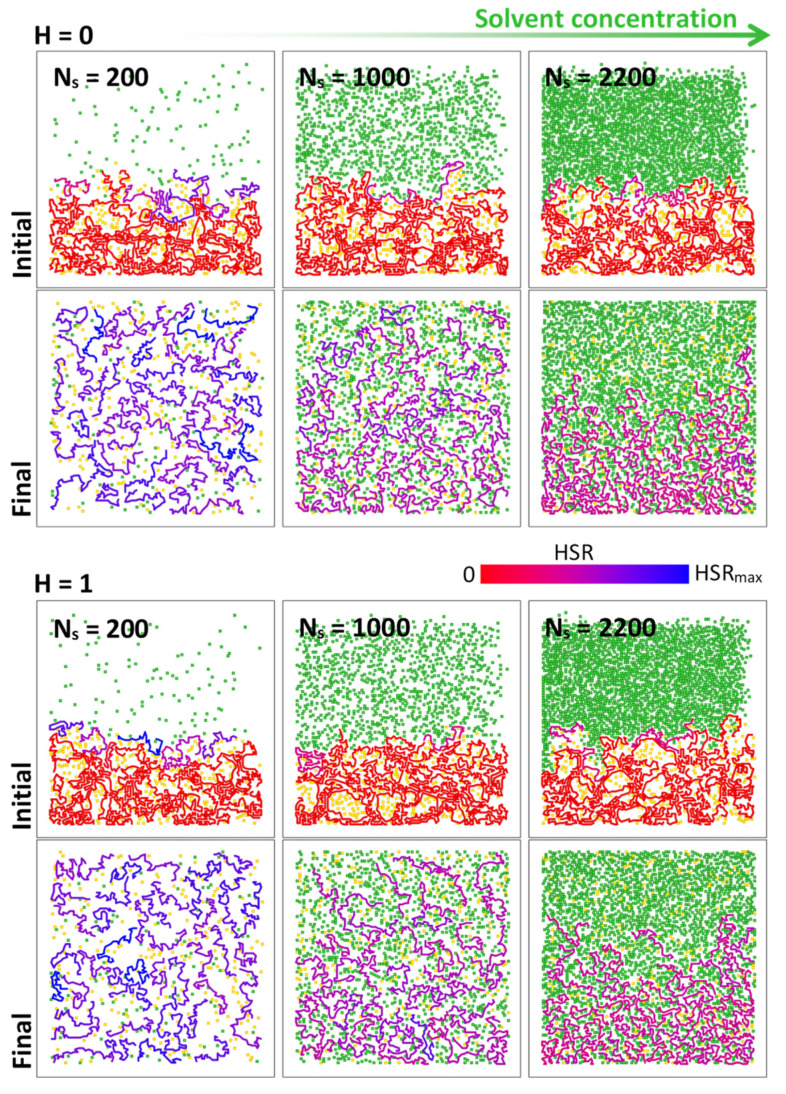
The effect of interaction energies (H=0, 1) and solvent density ($N_{s}=200,\;1000,\;2200$) on the polymer matrix swelling pattern. The polymer chains are colored according to the hit-by-solvent rate, HSR, with $HSR_{\max}=50/10^{5}$. The solvent and drug particles are colored in green and yellow, respectively.

Figure 3 depicts the initial and final tablet swelling snapshots, obtained with two different sets of interaction energies (H=0, 1) and three different solvent densities ($N_{s}=200,\;1000,\;2200$). In the snapshots, the initially immobile polymer chains are colored in red, while the mobile chains are colored bluish according to the current value of the hit-by-solvent rate, HSR (i.e., the number of the solvent–chain NN contacts in 10^5^ attempted MC steps). The solvent and drug particles are colored in green and yellow, respectively. Four features stand out from the snapshots. First, due to the stochastic nature of the tablet generation process, the initial tablet structures can differ by the porosity pattern (pore size and pore spatial distribution) that is formed during the sedimentation and the pore formation processes. This can result in different drug loadings and, hence, to some extent, also in altered swelling dynamics. Second, the polymer matrix swelling process runs gradually in the form of chain disentanglement and the removal of individual polymer chains from the polymer matrix into the surrounding solvent. Third, within the frame of 2D BFM, solvent density plays an important role. While small $N_{s}$ implies a relatively smaller chain–solvent interaction cross-section that is further associated with the solvent-mediated chain activation rate at the initial stages, it also results in a reduced interaction cross-section of the activated chains traversing through the solvent medium. With an increasing $N_{s}$, the effect of the interactions (steric and NN) progressively prevails over the enhanced initial chain activation rate. The hindered chain dynamics are especially apparent in the final snapshots, in which the activated chains populate only the lower half of the simulation box. Fourth, with the relatively small BFM numbers used in this study, the interaction energies (H) have only a minor effect on the final distribution of the activated polymer chains.

**Figure 4:**
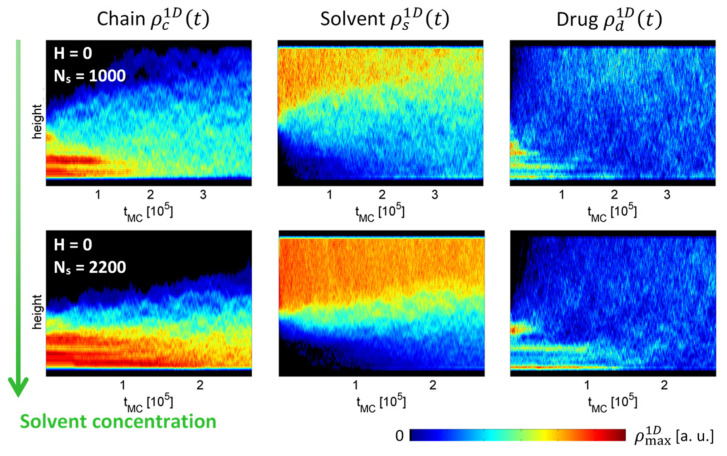
One-dimensional density profiles of swelling polymer, penetrating solvent, and released drug BFM particles as a function of time. The profiles were obtained by using the 2D BFM simulations with H=0 and two different solvent densities ($N_{s}=1000,\;2200)$.

One-dimensional vertical density profiles of all three species (chains, solvents, and drugs) as a function of time for two different solvent densities ($N_{s}=1000,\;2200$) and H=0 are shown in Figure 4. The density profiles correspond to the initial/final snapshots of medium ($N_{s}=1000$) and high ($N_{s}=2200$) solvent density with H=0 in Figure 3. As can be seen from the density profiles, increased solvent density results in slowed polymer matrix dynamics, as well as hindered penetration of solvent particles into the polymer matrix interior. Albeit the simulation times differ (approximately for a factor of one-half on account of two-fold larger $N_{s}$), the profiles obtained with different $N_{s}$ exhibit significantly different time courses.

**Figure 5:**
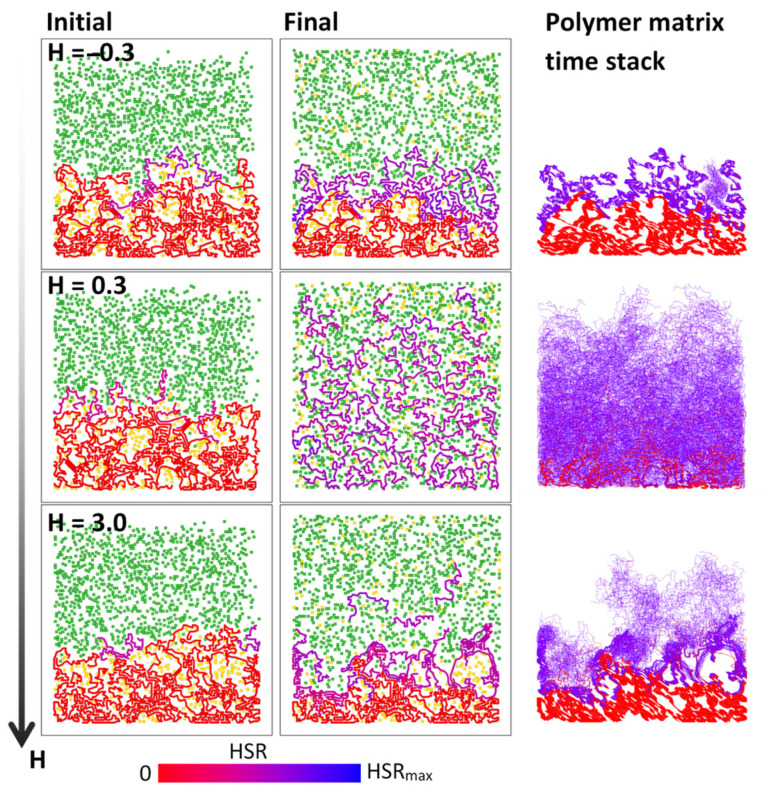
The initial (**left**) and final (**middle**) snapshots of dissolving polymer matrices, obtained by 2D BFM simulations with interaction energies H=−0.3, 0.3, 3 and solvent density $N_{s}=1000$, as well as the corresponding polymer matrix time stacks (**right**), each with the superposition of 50 polymer matrices equidistant in simulation time between the initial and the final polymer matrix structure. Color of chains varies depending on hit-by-solvent rate (HSR). The solvent and drug particles are colored in green and yellow, respectively.

The effect of the pairwise interaction energies (for three different H=−0.3, 0.3, 3) on the swelling of the polymer matrix with $N_{s}=1000$ is shown in Figure 5, which depicts the initial (left column) and final (middle column) snapshots with all three involved species, as well as a time stack of the polymer matrix corresponding to the superposition of 50 snapshots of the polymer matrix at equidistant simulation times between the start and end of the simulation (right column). As can be seen, extending H values below zero and beyond unity has a pronounced effect on polymer matrix swelling. Polymer matrix swelling is the fastest with H=0.3, i.e., implying repulsive interaction energies $E_{cc}>0$ and $E_{cs}>0$, which results in the gradual disentanglement of polymer chains that are followed by chain accommodation over the entire available space. With H=−0.3, contacts between chains and solvent particles that initialize the chain dynamics are relatively rare due to the repulsive chain–solvent interaction ($E_{cs}>0$). Also, when the chains are activated, the chain-activating solvent particles tend to abandon the chain–solvent NN interaction zone. On the other hand, the chain–chain energy is negative, $E_{cc}<0$, which, in turn, results in attraction between the neighboring chains. This also prevents further swelling dynamics. With H=3, the swelling dynamics are also impeded, however the interaction energies are with the opposite signs, i.e., $E_{cs}<0$ and $E_{cc}>0$. The attractive chain–solvent interaction promotes establishing chain–solvent contacts that also activate chain dynamics. The repulsive chain–chain energy would result in polymer matrix expansion. However, the chain dynamics are hindered by the attractive chain–solvent interaction that prevents the removal of solvent particles from the chain NN vicinity. The impeded chain dynamics can be additionally seen from the polymer matrix time stacks in the right column of Figure 5, in which the significant dynamics are obtained only with repulsive chain–solvent and chain–chain interactions (the case with *H* = 0.3).

**Figure 6:**
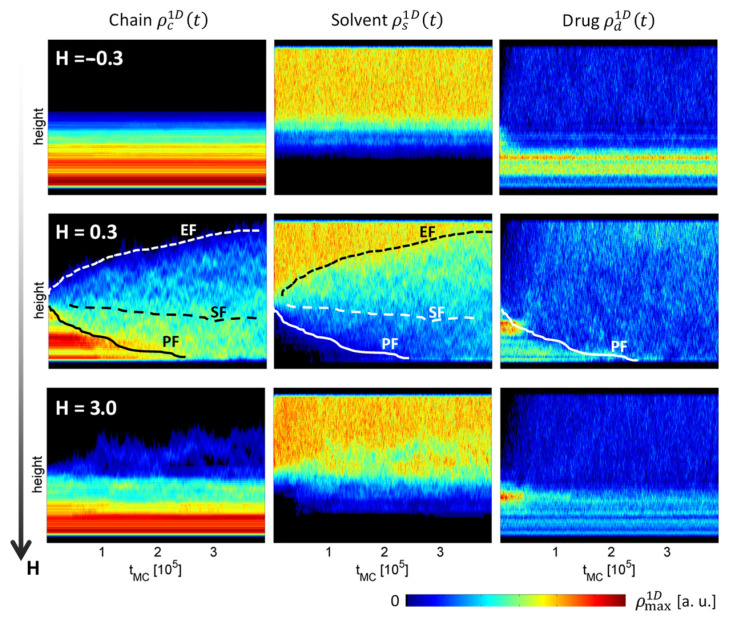
One-dimensional density profiles of all three species (polymer chains, solvents, and drug particles) as a function of time. The profiles were obtained by 2D BFM simulations with interaction energies H=−0.3, 0.3, 3 and solvent density $N_{s}=1000$. The curves schematically denote penetration (PF, solid), swelling (SF, dashed), and erosion (EF, dotted) fronts.

The effect of interaction energies (H=−0.3, 0.3, 3) on time-dependent 1D density profiles of all three involved species is shown in Figure 6. The profiles correspond to the polymer matrix structures presented in Figure 5. Polymer matrix swelling is prevented completely (H=−0.3) or partially (H=3), whereas the swelling process gradually progresses with the intermediate interaction energy parameter (H=0.3), as can be best seen from the polymer chain density profiles. The profile clearly exhibits four distinct regions separated by three transition fronts: first, between the yet immobile bulk polymer matrix and the partially swollen polymer matrix (solid line, PF—penetration front); second, between the partially swollen polymer matrix and individual highly mobile polymer chains (dashed line, SF—swelling front) and; third, between the highly mobile polymer chains and the solvent medium (dotted line, EF—erosion front). All three fronts are also clearly discernible from the complementary solvent density profile, while only two distinct regions are found in the drug profile. The two densities, high density and low density, correspond to drug particles entrapped in the polymer matrix pores and released drug particles, respectively.

**Figure 7:**
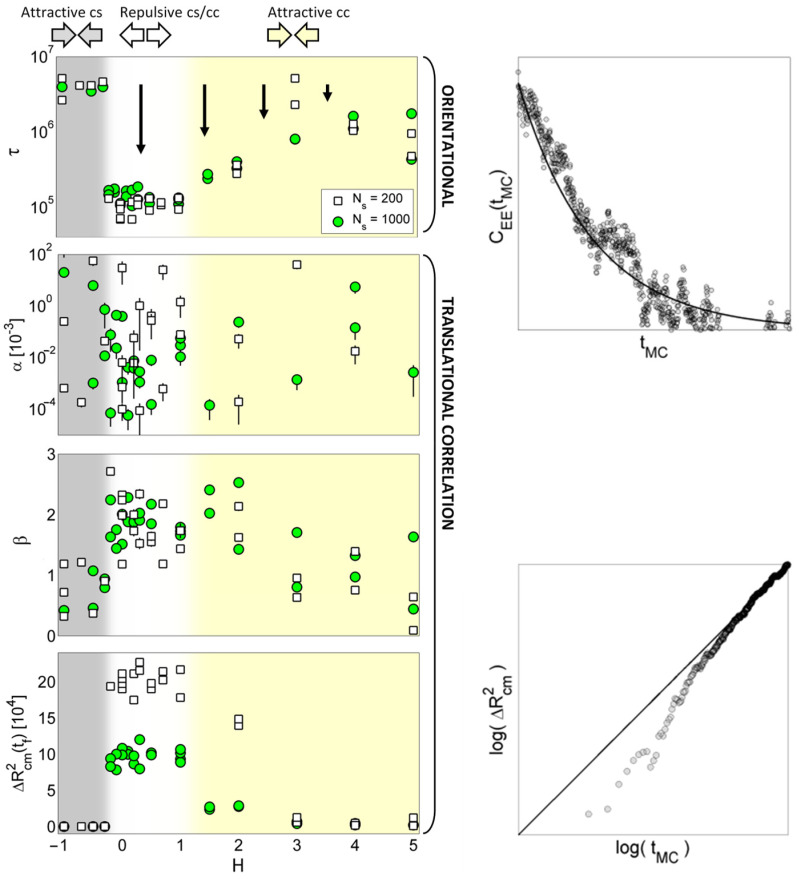
The fitting parameters τ, α, and β (Equations (3) and (4)), along with $\Delta R_{cm}^{2}\left(t_{f}\right)$ as a function of interaction energies (−1≤H≤5) and solvent density ($N_{s}=200,\;1000$), as well as two side plots depicting representative orientational (Equation (1)) and translational (Equation (2)) correlation functions. The H parameter defines three different interaction regimes in the graphs, denoted by the colored areas. The black vertical arrows emphasize the direction of the changes in the plotted parameters.

Figure 7 summarizes the results of the quantitative analysis of the 2D BFM simulations (total of 62 simulation runs) for a range of interaction energies −1≤H≤5 and for two different solvent densities ($N_{s}=200,\;1000$). Specifically, the results show the fitting parameters of Equations (3) and (4), τ, α, and β, which were obtained from the calculated orientational (Equation (1)) and translational correlation functions (Equation (2)), as well as from the translational correlation function evaluated in at least two different simulation times.

With respect to these parameters, three different chain motional regimes can be observed in the graphs of Figure 7. With H≲−0.2, the attractive chain–solvent interaction results in the motional frustration of polymer chains that is manifested by large orientational correlation times ($\tau \approx 5\times 10^{6}$), small translational exponents (β≈1), and $\Delta R_{cm}^{2}\left(t_{f}\right)=0$. In the range of −0.2≲H≲1.0, the interaction energies are positive and, thus, repulsive; therefore, the chain motion is not frustrated, as demonstrated with shorter orientational correlation times ($\tau \approx 1\times 10^{5}$) and faster translational motion (β≈2 and $\Delta R_{cm}^{2}\left(t_{f}\right)≳1\times 10^{5}$). With H≳1.0, the chain motion becomes gradually frustrated and the motional parameters gradually attain comparative values as with H≲−0.2. The effect of solvent density is most pronounced in the range of repulsive interactions (−0.2≲H≲1.0), where $\Delta R_{cm}^{2}\left(t_{f}\right)$ values are decreased with an increasing solvent density (green circles vs. white squares), while orientational correlation times are not dependent on solvent density. Also, faster translational dynamics with smaller $N_{s}$ are demonstrated with comparatively large values of $\Delta R_{cm}^{2}\left(t_{f}\right)\approx 2\times 10^{5}$. In Figure 7, the dependence of the parameter α on H is less apparent. An alternative, exploratory approach to analyze the differences between the orientational correlation functions and between the translational correlational functions upon various values of the parameters $N_{s}$ and H is a principal component analysis (PCA).

## 4. Discussion

In this presented work, polymer matrix tablet swelling was studied both experimentally by means of magnetic resonance microscopy (MRM), as well as numerically within the 2D BFM framework. The aim of the work was to elucidate the effect of the polymer–solvent interactions on the swelling process of polymer-based drug delivery systems. Experimentally, it was confirmed that MRM is an efficient method for a noninvasive and tomographic follow-up of the xanthan polymer matrix tablets during solvent-mediated swelling. The process was quantified in terms of three moving fronts (penetration, swelling, and erosion).

In order to obtain a deeper insight into the tablet swelling process, a 2D mathematical model of polymer chain matrix swelling within the BFM framework was developed. An apparent advantage of the BFM model is that it realistically captures the microscopic heterogeneity of the polymer matrix and, with it, the associated relevant features that govern the polymer matrix swelling process. Namely, the swelling process of the tablet structure runs in the form of the swelling of individual polymer chains, or fragments constituted of individual chains, that escape from the surface of the bulk polymer [4]. This is in contrast to the first mechanistic MC-based tablet disintegration models [19,40], in which the polymer matrices were approximated by rigid lattices, thus neglecting the chain-like nature of the constituting polymers. Moreover, an important BFM feature is also a self-avoidance that is imposed by strongly repulsive steric interactions. The BFM model thus captures polymer chain dynamics along the primitive paths that are strongly influenced by topological constraints, imposed by the surrounding medium [41]. Also, the interacting BFM particles (steric and NN) are simultaneously subjected to fierce competition for the available space. The competition can be, for example, visually clearly demonstrated in the density profiles of the most abundant species (i.e., polymer chain and solvent) that appear complementary (Figure 4 and Figure 6).

In this study, distinct swelling patterns were obtained by varying two pairwise interactions, $E_{cc}$ and $E_{cs}$, that were interdependent via the H parameter [29]. Its variation in a relatively large range also yielded some swelling patterns that cannot be straightforwardly compared with the experimental results. Upon contact with water molecules, a xanthan polymer matrix was transformed into a hydrogel by additional water-mediated cross-linking between the xanthan chains [4]. In the BFM model, however, positive interactions can imply the onset of motional frustration. In order to avoid motional frustration, therefore, the explicit-solvent BFM models are typically applied with positive interaction energies [29,32]. In terms of the solvent quality, i.e., a good solvent, in which the chains traverse freely, and a bad solvent, in which the chain motion is impeded due to attractive chain–solvent interactions, Lappala et al. recently demonstrated that the good-to-bad quality transition in long chains results in a raindrop-like coalescence of initially fully unfolded chains [42].

In order to keep the BFM simulations of polymer matrix swelling computationally feasible on a single desktop PC, the BFM model was implemented only in two dimensions so that it could involve a relatively small number of the BFM particles. This also partially explains the scatter of the fitting parameters in Figure 7. With an increasing number of BFM particles, it is expected that the scatter, as well as the effect of the initial stochastically generated polymer matrix structure on the swelling process, would be remarkably reduced. Moreover, larger BFM systems would also make the simulation results in better accord with the MRM results (mesoscopic spatial scales with a voxel size of 100 μm), as well as unveil possible differences in motional correlation parameters in the repulsive-energy range (0≲H≲1). These differences are expected to better explain the experimentally obtained differences in solvent-dependent swelling dynamics. This study also reveals an interesting behavior of the parameter β from values larger than two to lower than one as the parameter H increases (Figure 7), which means that the system displays complex dynamics. For β>2, the motion is “stronger” than ballistic, while the change from β>1 to β<1 corresponds to the change from superdiffusion to subdiffusion, which is an interesting effect in the complex dynamics of polymers [43]. Polymer swelling kinetics can also be evaluated at the macroscopic level by measuring the swelling content $S=\left(w_{s}-w_{0}\right)/w_{0}$ as a function of time and analyzing it with a swelling kinetics model, e.g., with the first-order model $S\left(t\right)=S_{eq}\left(1-e^{-kt}\right)$ [44,45,46]. Here, $w_{s}$ and $w_{0}$ are the swollen and initial tablet weights, respectively, and $S_{eq}$ is the equilibrium swelling content. An analysis of the sequential MRI images (Figure 2) of xanthan polymer matrix tablet hydration yielded the parameters of this model equal to $S_{eq}=7.7$ and $k=0.022\;h^{-1}$.

The polymer–solvent system is usually studied by the Flory–Huggins (polymer–solvent) interaction parameter $\chi_{12}$ [47]. This can be calculated from the molecular interactions between solvent–solvent $w_{11}$, monomer–monomer $w_{22}$, and monomer–solvent $w_{12}$ pairs, and from the coordination number z, which is equal to the number of interacting nearest-neighbors as $\chi_{12}=z\left(w_{12}-\frac{1}{2}\left(w_{11}+w_{22}\right)\right)/\left(k_{B}T\right)$, where $k_{B}$ is Boltzmann’s constant. For the model in this study, the molecular interaction parameters are $w_{11}=0$, $w_{22}=\varepsilon_{0}H$, and $w_{12}=\varepsilon_{0}\left(1-H\right)$, and the coordination number is z=24, so the polymer–solvent interaction parameter is equal to $\chi_{12}=24\varepsilon_{0}\left(1-\frac{3}{2}H\right)/\left(k_{B}T\right)$. Since $\varepsilon_{0}=0.8$ is constant and temperature T is not used as a model parameter, the only changing parameter in $\chi_{12}$ is the interaction energy (relative hydrophobicity) *H*, with which $\chi_{12}$ is linearly proportional.

Some experimental studies [48,49] highlighted the importance of the solvent environment for the gradual swelling of the xanthan tablet and the associated controlled release of the drug compound. The swelling process was experimentally found to be dependent on the solvent–polymer interaction that was provided by different values of ionic strength and pH of the solvent environment. Specifically, the swelling process was faster with a water solvent environment, while the process was slowed with the HCl acidic solvent environment. Another BFM limitation is the onset of steric quenching in the model, but this effect is not expected to be detected experimentally either. Moreover, a variation of the H parameter roughly defines three different interactions and, thus, also swelling regimes (as seen in Figure 7). In this study, the presented simulation tools were verified only on one experimental example. In future studies, we plan to validate the model on several different polymer–solvent pairs, especially those used for controlled-release tablets.

## 5. Conclusions

This study presents a combined experimental (xanthan tablet swelling nondestructively followed by MRM) and numerical study of polymer matrix swelling (employing 2D BFM) in order to address the effect of polymer–solvent interactions on the evolution of the swelling process. Although the spatial and temporal scales in the experiments and numerical simulations are not directly comparable, this study represents a combined approach to address polymer–solvent interactions in optimizing controlled drug release from polymer matrix tablets.

## Data Availability

The data presented in this study are available upon request from the corresponding author.
